# Reducing Cranial Morphology Error in F3 Scalp Localization: Comparison of Heuristic and Regression‐Based Methods

**DOI:** 10.1002/brb3.71805

**Published:** 2026-10-08

**Authors:** Rosa Pàmies‐Vilà, Laia Mallol‐Bordas, Vicenç Pascual‐Rubio, Albert Fabregat‐Sanjuan

**Affiliations:** ^1^ Department of Mechanical Engineering and Institute for Research and Innovation in Health Universitat Politècnica de Catalunya—Barcelona Tech Barcelona Spain; ^2^ Department of Mechanical Engineering Universitat Rovira i Virgili Tarragona Spain; ^3^ Department of Clinical Neurophisiology and Hospital Universitari Sant Joan de Reus, NeuroÈpia Southern Catalonia Biomedical Research Institute – IRB CatSud Reus Spain

**Keywords:** Beam‑F3 method, cranial morphology, F3 localization, non‑invasive brain stimulation, rTMS targeting, scalp‑based localization

## Abstract

**Purpose:**

Accurate localization of the F3 scalp position is essential for targeting the dorsolateral prefrontal cortex in repetitive transcranial magnetic stimulation (rTMS), particularly when neuronavigation is unavailable. However, simplified scalp‐based heuristic methods may lead to clinically relevant targeting errors due to substantial interindividual variability in cranial morphology. In this context, this study evaluates the geometric accuracy of commonly used and newly proposed scalp‐based approaches for localizing the F3 position within the International 10–20 system. Specifically, it examines the impact of cranial morphological variability on localization error and assesses whether individualized regression‐based methods can improve targeting accuracy compared to traditional heuristic techniques.

**Method:**

The intrinsic geometric accuracy of five F3 localization methods was systematically compared: BeamF3, Adjusted BeamF3, the Scalp Geometry‐based Parameter (SGP) method, NeurallyF3, and a newly developed approach EPlacementF3. A total of 250 anatomically realistic and morphologically diverse 3D head models (125 female, 125 male), generated using the HumanShape platform, were analyzed. Localization error was quantified as the Euclidean distance between each estimated F3 position and the reference coordinate defined by the International 10–20 system.

**Findings:**

BeamF3 and Adjusted BeamF3 showed the highest localization errors (mean >5.5 mm and >8 mm, respectively), while the SGP method improved accuracy (≈2.4 mm). In contrast, NeurallyF3 and EPlacementF3 achieved submillimetric errors across sexes. EPlacementF3 demonstrated the best overall performance, with the lowest mean error (∼0.2 mm) and consistent results in the independent validation dataset. These findings highlight the strong influence of cranial morphology on heuristic methods and the superior robustness of individualized regression‐based approaches.

**Conclusion:**

Commonly used heuristic methods do not reliably recover the true F3 position across heterogeneous cranial morphologies. In contrast, individualized regression‐based approaches substantially reduce morphology‐related error, supporting more accurate and scalable MRI‐free targeting for rTMS and other non‐invasive brain stimulation applications.

## Graphical abstract

We systematically evaluated the intrinsic geometric accuracy of five F3 localization methods in 250 3D head models (125 female and 125 male). Commonly used heuristic approaches (BeamF3, Adjusted BeamF3, and SGP) showed higher errors, whereas regression‐based methods (NeurallyF3, EPlacementF3) achieved submillimetric accuracy, enabling reliable MRI‐free rTMS targeting.

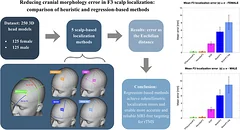

## 1 Introduction

Accurately identifying specific cranial landmarks is crucial for both research and clinical applications of non‐invasive brain stimulation (NIBS). A key target in this field is the left dorsolateral prefrontal cortex (DLPFC), a brain region that supports executive functions, including working memory, cognitive flexibility, and top–down control of attention and behavior. This area is frequently selected for interventions such as repetitive transcranial magnetic stimulation (rTMS) for major depressive disorder (Avery et al. 2006; McClintock et al. 2018; Seibt et al. 2015). However, due to the natural anatomical variability in human cranial morphology (Jurcak et al. 2007; Scrivener and Reader 2022), consistently locating precise stimulation sites remains a challenge.

To address this, the International 10–20 System (IS 10–20), introduced by Jasper (1958)provides a widely accepted framework for standardizing the positioning of 21 scalp locations based on four universal cranial landmarks: the nasion, inion, and the left and right preauricular points (Tragus or LPA and RPA). The F3 position from the IS 10–20 serves as the anatomical guide for coil placement when DLPFC is targeted (Mir‐Moghtadaei et al. 2015). However, the manual standard localization protocol for F3 involves up to 19 measurement steps using tools like measuring tape and skin markers, which increases preparation time and introduces opportunities for human error.

To simplify this process, heuristic methods such as BeamF3 (Beam et al. 2009) and its adjusted version have been developed and are widely used in clinical practice. Nevertheless, recent studies have highlighted discrepancies between heuristic‐based estimations and the standard IS 10–20 (Fabregat‐Sanjuan et al. 2022). Such inconsistencies may lead to suboptimal targeting of the DLPFC, potentially reducing treatment efficacy. This may partially explain the substantial variability in patient response to rTMS for major depressive disorder, where only 40%–50% of individuals with treatment‐resistant depression achieve a clinically meaningful response (George et al. 2010). One hypothesis for this variability is that the resolution of the defined target site could play an important role in determining rTMS outcomes (George et al. 2010; Herbsman et al. 2009).

A new alternative for BeamF3 has been proposed to locate the F3 position, the scalp geometry‐ based parameter (SGP) system, introduced by Jiang et al. (2022). However, SGP relies on MNI coordinates or an averaged F3 position rather than individual measurements. Moreover, SGP changes the methodology to find F3 position from *X* and *Y* coordinates from BeamF3 method, being more difficult to be applied by clinicians. A recent study has compared the accuracy of BeamF3 and SGP (Li et al. 2025), showing higher accuracy for SGP method. However, the study relied on head models with limited variability in head circumference (HC) and morphology, providing am inadequate representation of real‐world anatomical diversity.

To address the inaccuracies of existing methods, our research group, focused on advancing clinical neurophysiology, has developed two new approaches for locating the F3 point that include real‐word head morphology diversity. NeurallyF3 (Pàmies‐Vilà et al. 2026) was previously developed and validated as an adaptation of the BeamF3 procedure, following the same measurement steps but changing the values of *X* and *Y*, to 𝑋_Neurally‐F3_ and *Y*_Neurally‐F3._ The second method, EPlacementF3, is introduced in the present study as a novel approach derived from our previously described EPlacement system (Fabregat‐Sanjuan et al. 2023), which can be used in combination with a medical device to simplify the implementation and allows not only the location of F3 position but all positions from IS 10–20 and 10–10 (Fabregat Sanjuan and Pascual Rubio 2023).

EPlacementF3 uses a different geometric approach designed to simplify clinical implementation. Unlike NeurallyF3, EPlacementF3 does not require transferring a measurement along the HC (errors can be made identifying FPz and Oz), and measurements can be taken from nasion. EPlacementF3 simplifies the clinical procedure while maintaining an individualized geometric approach that accounts for inter‐individual differences in head morphology.

The objective of the present study is to systematically compare the geometric accuracy of five scalp‐based methods for localizing the F3 position—namely, the standard BeamF3 method, its adjusted variant, the SGP approach, the previously developed NeurallyF3 method, and our newly proposed EPlacementF3 method—using a morphologically diverse dataset of anatomically realistic cranial models. By quantifying the Euclidean localization error associated with each approach, we aim to evaluate their robustness in the presence of interindividual cranial variability and to determine the extent to which morphology‐dependent error can be reduced.

Based on the documented influence of cranial morphology on proportional localization systems, we hypothesize that regression‐based methods incorporating multiple individualized anthropometric parameters will achieve significantly lower geometric error and variability than traditional heuristic approaches that rely on fixed proportional assumptions.

## 2 Methods and Materials

### 2.1 Dataset and Head Shape Models

To obtain a sample of anatomically realistic human heads, the HumanShape head‐shaped model (University of Michigan Transportation Research Institute (UMTRI) 2020) has been used. This model developed by the University of Michigan is based on statistical analysis of high‐resolution laser scans of 100 women and 80 men to create a model with 58 reference points that create a mesh to ensure anatomical consistency of the scan data (Park et al. 2021). The HumanShape web platform allows the generation of 3D models of male and female heads by adjusting four parameters: HC, breadth to length ratio, bitragion chin arc, and tragion to top distance. The influence of the bitragion chin arc parameter is irrelevant, as it modifies the morphology of the lower part of the face without affecting the area of the IS 10–20. Therefore, it is not used and is set to the mean value.

For this study, two independent datasets were created. The development dataset consisted of 250 head models (125 female and 125 male), generated using a design of experiments (DOE) approach. Three key anthropometric parameters—HC, breadth‐to‐length ratio, and tragion‐to‐top distance—were systematically varied across the fifth, 25th, 50th, 75th, and 95th percentiles, ensuring broad coverage of cranial morphologies. This dataset was used to develop the EPlacementF3 models (male and female). The same 250 heads were also used to evaluate the heuristic methods SGP, BeamF3, and BeamF3 Adjusted, providing a broad comparative error analysis.

An independent validation dataset comprising 128 additional head models (64 female and 64 male) was generated using intermediate percentiles (15th, 37th, 63rd, and 85th), resulting in morphologies not included in the development dataset. This independent set was used to assess the generalizability and robustness of EPlacementF3 on unseen data. Overall, the 378 head models covered a wide range of head shapes and sizes representative of real populations while preserving a clear separation between model development and independent validation.

### 2.2 EPlacementF3 Method

The EPlacementF3 method introduces a novel approach for accurately locating the F3 scalp position, based on proportional distances along anatomically relevant reference lines. The development of the method involved a training dataset of 250 realistic head models in which the exact F3 position was known.

For each head model, two key percentages were computed (Figure 1):

‐ *Cz to C3*′: the percentage distance from the vertex point Cz to an intermediate point C3′, measured along the tragus–tragus (Tr–Tr) line.
‐ *Nasion to F3*: the percentage distance from the Nasion to the F3 position, measured along an inclined arc extending from the Nasion to the Inion, passing through the previously defined C3′ point.

**FIGURE 1:**
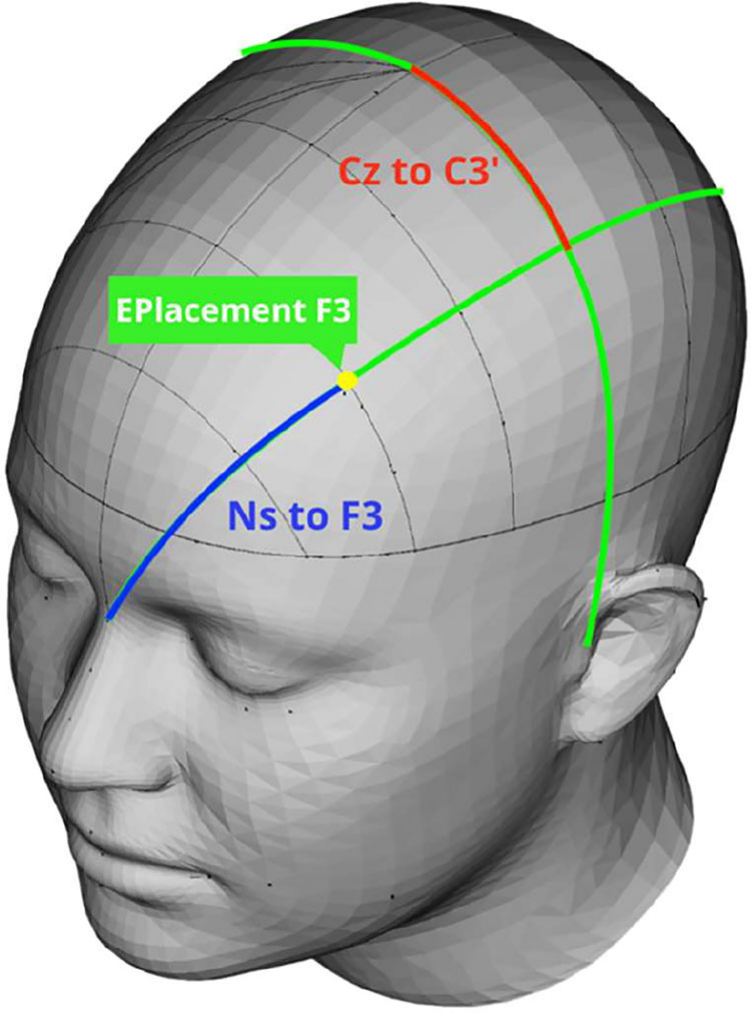
EPlacementF3: Distances Cz to C3′(in red) and Ns to F3 (in Blue).

These percentages were first computed individually for all 250 head models thanks to a developed script in Python that can perform all the steps needed in FreeCAD to calculate the F3 position and the length of the percentages. Based on these data, a multiple linear regression model was developed using three anatomical measurements as predictors: the nasion–inion distance, the tragus–tragus distance, and the HC. This model allows us to predict the proportional distances required to locate the F3 position in new individuals, provided their cranial dimensions.

To validate the methods, all five approaches (EPlacementF3, NeurallyF3, SGP, BeamF3, and BeamF3 Adjusted) were subsequently evaluated using the testing dataset consisting of 128 additional head models (64 female and 64 male) that were not used during the development of EPlacementF3 (training phase). For each head shape model, the same procedural steps outlined previously were followed, and the errors between the proposed F3 locations and the true F3 position (determined by the IS 10–20) on each model, were calculated to evaluate the accuracy. The localization error was quantified by calculating the Euclidean distance between the location of the estimated F3 and the location of the true F3 position.

### 2.3 F3 Localization Procedures

The head mesh models are obtained from the web and imported to the parametric 3D computer‐aided design (CAD) modelling application FreeCAD. Then, the procedures to obtain the International 10–20 System F3 point are implemented. To facilitate the process, a Python macro has been developed to automate the procedure.

Five different scalp‐based localization methods to locate the F3 point are studied (Figure 2). The BeamF3 and BeamF3 Adjusted methods (Beam and Borckardt 2010), the SGP method (fNIRS Brain Imaging and TMS Neuromodulation Group 2022), the previously developed and validated NeurallyF3 method (Clinical and Epidemiological Neuroscience (NeuroÈpia) Research Group 2025b), and the newly proposed EPlacementF3 method (Clinical and Epidemiological Neuroscience (NeuroÈpia) Research Group 2025a). The five methods were introduced into the script of the 3D design program to locate the approximations of point F3 of each method. On the cases of BeamF3 and SGP methods, the values provided by their websites to locate the F3 position were used.

**FIGURE 2:**
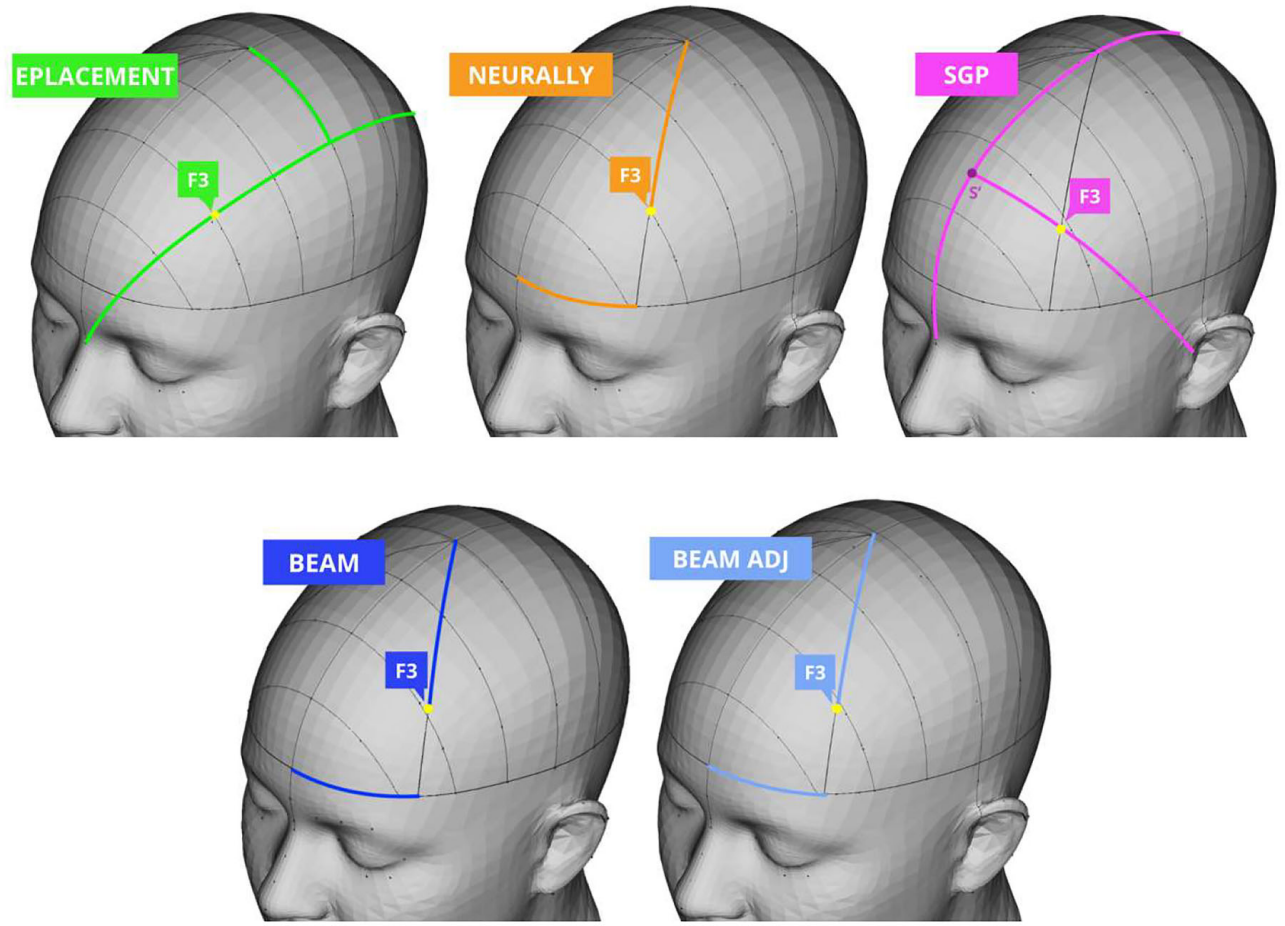
Methods to locate the F3 point: EPlacementF3, NeurallyF3, SGP, Beam, and Beam Adjusted.

## 3 Results

### 3.1 Localization Error Analysis of F3 Location

Applying the methodology described in the previous section, anthropometric measurements were obtained for the 250 head models used in the training phase (Table 1). These included HCs, nasion–inion distance (Ns–In), and tragus–tragus distance (Tr–Tr). To assess potential differences in cranial morphology between male and female subjects, a multivariate analysis of variance (MANOVA) was performed on these three variables. The results revealed a statistically significant multivariate difference between genders (*p* = 6.23 × 10^−^^2^ ^3^), prompting separate analyses for male and female subsets.

**TABLE 1:** Anatomical parameters of the 250 head models used separated by gender: HC, Ns–In length, and Tr–Tr length.

	Female			Male		
	Mean ± σ [mm]	Min [mm]	Max [mm]	Mean ± σ [mm]	Min [mm]	Max [mm]
HC	560.6 ± 22.1	515.3	606.95	573.4 ± 22.7	527.3	619.8
Ns–In	363.8 ± 20.9	314.1	412.95	362.6 ± 20.4	313.8	410.8
Tr–Tr	345.9 ± 23.1	301.5	391	345.5 ± 22.9	298.3	389.9
Ɛ EPlacementF3	0.20 ± 0.16	0.02	0.78	0.20 ± 0.14	0.01	0.63
Ɛ NeurallyF3	0.25 ± 0.17	0.03	0.81	0.298 ± 0.21	0.03	1.29
Ɛ SGP	2.39 ± 0.47	1.39	3.55	2.39 ± 0.54	1.12	3.36
Ɛ BeamF3	5.59 ± 1.23	3.33	8.16	6.09 ± 1.51	3.53	9.43
Ɛ BeamF3 Adjusted	8.12 ± 1.96	5.13	11.94	8.96 ± 2.16	4.95	13.24

*Note*: The table also reports the error associated with BeamF3, BeamF3 Adjusted, SGP, EPlacementF3, and NeurallyF3 localizations. Data are presented as mean ± standard deviation, along with minimum and maximum values for each parameter.

For each head, the location of the F3 point according to the International 10–20 system was established, along with its corresponding estimated positions using five heuristic methods: BeamF3, BeamF3 Adjusted, SGP, NeurallyF3, and EPlacementF3. The localization error (*ε*) for each method was calculated as the Euclidean distance between the estimated and reference F3 positions (Table 1).

To better illustrate these results, histograms were generated to visualize the error distribution across the 250 head models for each method (Figure 3). In addition, a bar chart was created to display the mean and standard deviation of the error for all five methods (Figure 4).

**FIGURE 3:**
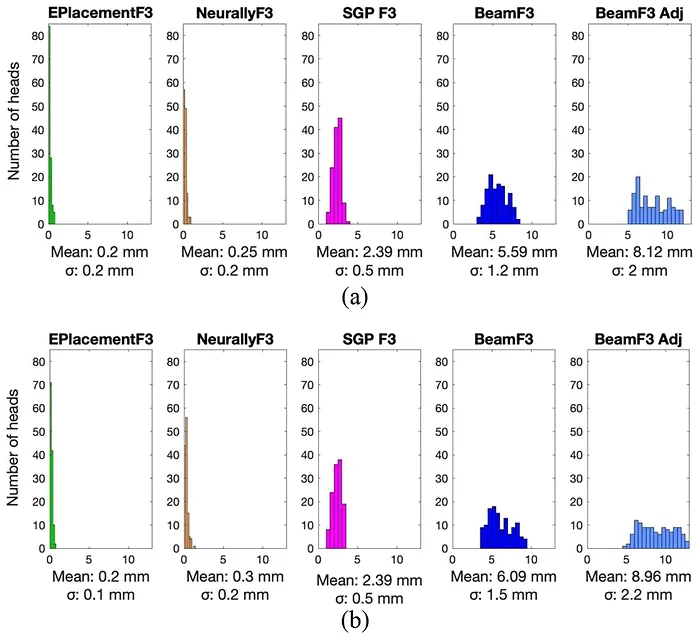
Histograms illustrating the distribution of localization error metrics for EPlacementF3, NeurallyF3, SGP F3, BeamF3, and Adjusted BeamF3 across the dataset, presented separately for female (a) and male (b) head models.

**FIGURE 4:**
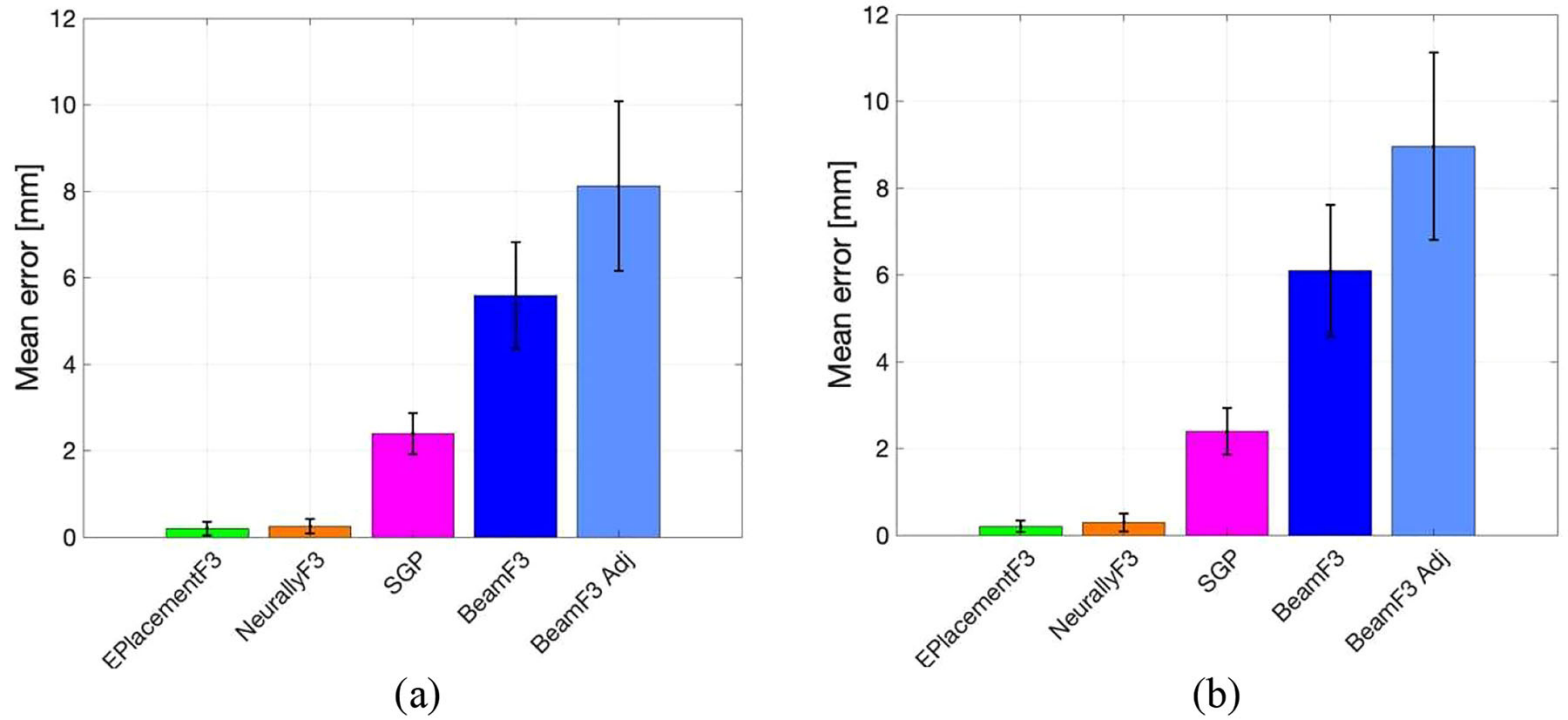
Bar chart displaying the mean localization error and standard deviation for each method, arranged in ascending order of error, separated by gender: female (a) and male (b).

To validate the newly proposed EPlacementF3 method, the second dataset comprising 128 independent head models—previously described in Section 2 and distinct from those used in the training phase—was analyzed. Table 2 summarizes the anthropometric data for this validation set, reporting the mean ± standard deviation, as well as minimum and maximum values for each parameter. For comparative purposes, localization errors for the NeurallyF3, SGP, BeamF3, and BeamF3 Adjusted methods are also included in this table.

**TABLE 2:** Anatomical parameters from the 128 head models used to validate the proposed model: HC, Ns–In and Tr–Tr, categorized by gender.

	Female validation			Male validation		
	Mean ± σ [mm]	Min [mm]	Max [mm]	Mean ± σ [mm]	Min [mm]	Max [mm]
HC	559.6 ± 18.1	523.8	595.7	572.1 ± 18.7	536.02	609.37
Ns–In	363.3 ± 17.2	324.6	401.8	361.8 ± 16.9	323.9	400.6
Tr–Tr	345.1 ± 19.4	308.2	381.4	344.9 ± 19.3	308	381.1
Ɛ EPlacementF3	0.16 ± 0.12	0.01	0.59	0.18 ± 0.11	0.03	0.47
Ɛ NeurallyF3	0.19 ± 0.11	0.01	0.59	0.23 ± 0.14	0.03	0.64
Ɛ SGP	2.33 ± 0.38	1.56	3.17	2.28 ± 0.46	1.4	3.09
Ɛ BeamF3	5.36 ± 1.0	3.63	7.26	5.96 ± 1.29	3.89	8.63
Ɛ BeamF3 Adjusted	7.98 ± 1.67	5.58	10.99	8.91 ± 1.85	5.85	12.35

*Note*: The table also reports the error associated with BeamF3, BeamF3 Adjusted, SGP, EPlacementF3, and NeurallyF3 localizations. Data are presented as mean ± standard deviation, along with minimum and maximum values for each parameter.

The same graphical analyses from training dataset were performed for the validation dataset (Figures 5 and 6). Results show a consistent trend across methods, with notably lower mean errors. This improvement can be attributed to the reduced variability in the second dataset, which does not include extreme morphological cases, resulting in a narrower range between minimum and maximum values.

**FIGURE 5:**
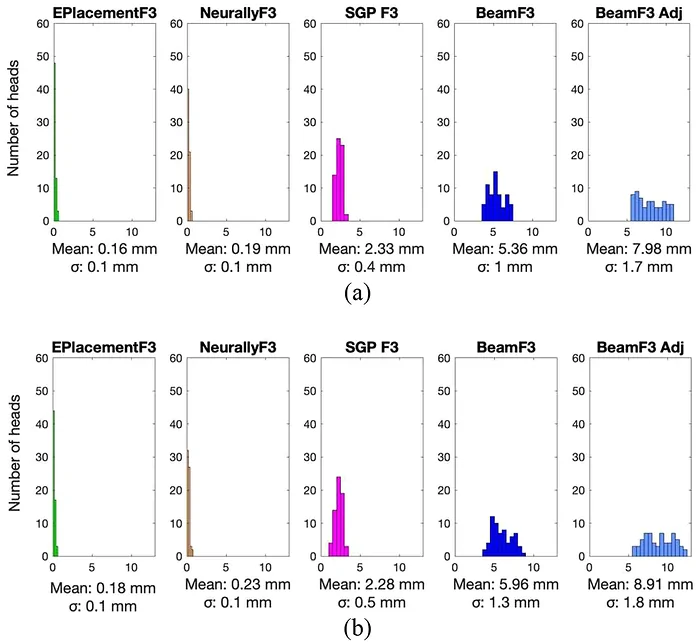
Histograms illustrating the distribution of localization error metrics for EPlacementF3, NeurallyF3, SGP, BeamF3, and Adjusted BeamF3 across the validation dataset, presented separately for female (a) and male (b) head models.

**FIGURE 6:**
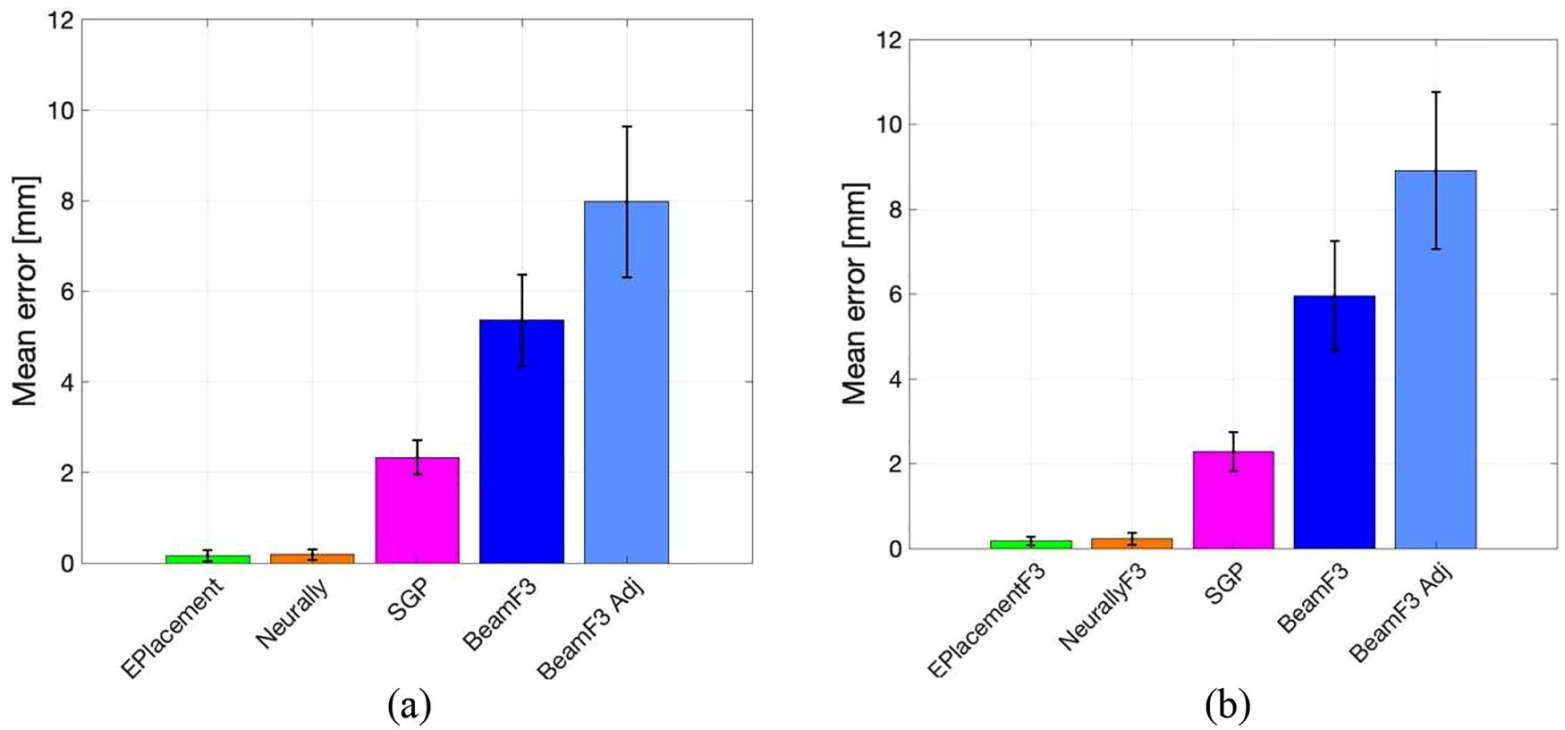
Bar chart displaying the mean localization error and standard deviation for each method on the validation dataset, arranged in ascending order of error, and separated by gender: female (a) and male (b).

It is important to emphasize that the EPlacementF3 model was developed using the initial set of 250 head models. As a result, its error values in this dataset are expected to be lower. This is precisely why validation with a completely independent set of 128 heads is essential—to assess the generalizability and robustness of EPlacementF3 when applied to unseen data. Nevertheless, the full dataset of 250 heads was used to evaluate the performance of the other heuristic methods (SGP, BeamF3, and BeamF3 Adjusted) and NeurallyF3 to ensure a broader and more comprehensive error analysis.

### 3.2 Variability of EPlacementF3 Method as a Function of Cranial Morphology

The EPlacementF3 method allows the percentage values (%Ns to F3 and %Cz to C3’) to be adapted to each individual rather than relying on a single average percentage for all subjects. To justify this individualized strategy, multiple linear regression plots were analyzed to assess how each parameter influences the calculated percentages. In the following plots (Figures 7, 8, 9, 10), the green lines show the partial effects estimated by the multiple linear regression, reflecting the influence of each variable while accounting for the effects of the other variables. The results illustrate the contribution of each anatomical measurement (HC, Ns–In, Tr–Tr) and support the need for a personalized estimation rather than a mean‐based approximation.

**FIGURE 7:**
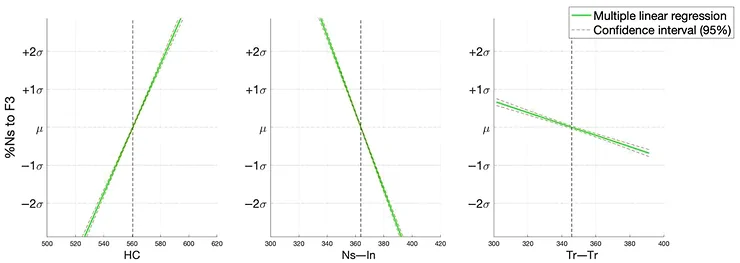
Multiple linear regression of %Ns to F3 as a function of HC, Ns–In, and Tr–Tr, centered at the mean of each parameter. The *Y*‐axis is scaled from ‐2σ to + 2σ around the mean percentage—Female model.

**FIGURE 8:**
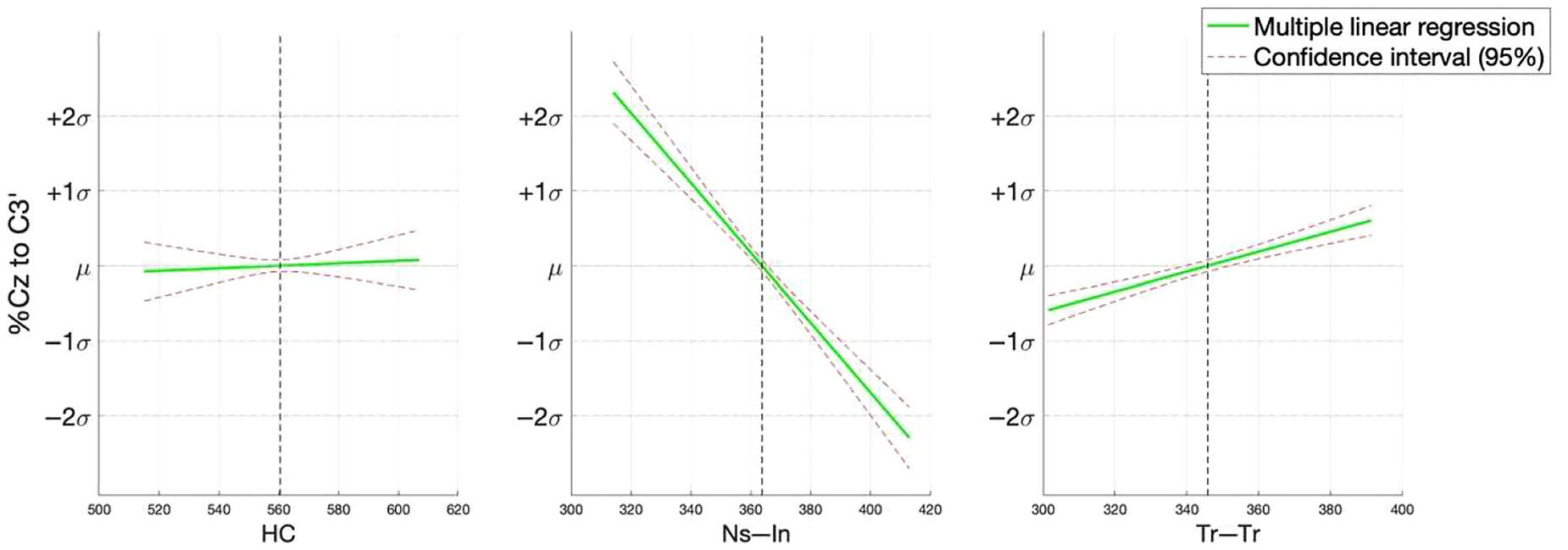
Multiple linear regression of %Cz to C3’ as a function of HC, Ns–In, and Tr–Tr, centered at the mean of each parameter. The *Y*‐axis is scaled from ‐2σ to + 2σ around the mean percentage—Female model.

**FIGURE 9:**
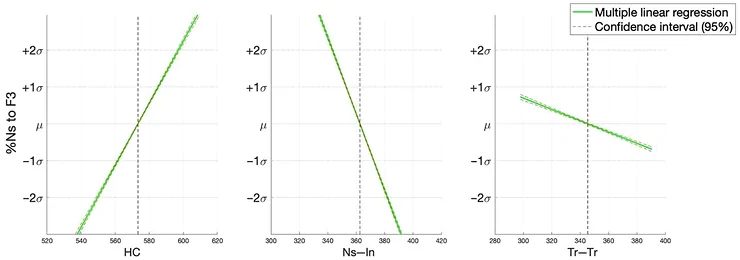
Multiple linear regression of %Ns to F3 as a function of HC, Ns–In, and Tr–Tr, centered at the mean of each parameter. The *Y*‐axis is scaled from ‐2σ to + 2σ around the mean percentage—Male model.

**FIGURE 10:**
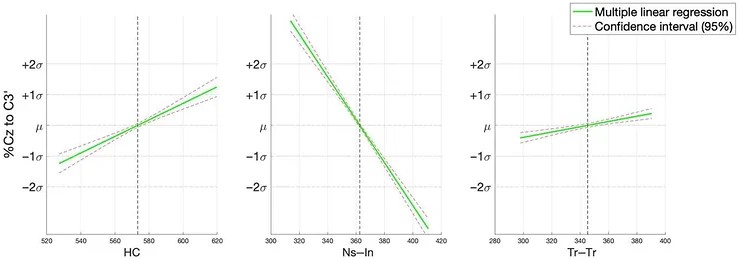
Multiple linear regression of %Cz to C3’ as a function of HC, Ns–In, and Tr–Tr, centered at the mean of each parameter. The *Y*‐axis is scaled from ‐2σ to + 2σ around the mean percentage—Male model.

As can be observed in the previous graphs, for the %Ns to F3, a larger HC combined with a smaller Ns–In distance results in a higher percentage value. In contrast, the Tr–Tr measurement has a smaller influence on the calculation; however, a slight tendency toward a decrease in the percentage can be observed as the Tr–Tr distance increases.

For the %Cz to C3’, a similar effect is observed for HC and Ns‐In, although the slope associated with HC is less pronounced, indicating a lower influence. Moreover, HC appears to have a stronger influence in males than in females. The Tr–Tr parameter also shows a minor effect, but in this case a slight increase in the percentage is observed as Tr–Tr increases.

## 4 Discussion

This study systematically evaluated the positional error associated with several heuristic methods for localizing the F3 point within the International 10–20 system: BeamF3, BeamF3 Adjusted, the SGP method, NeurallyF3, and the newly proposed EPlacementF3. The analysis was conducted on a large dataset of 3D head models to ensure robust comparisons under controlled conditions.

It is important to acknowledge that the reported errors were derived from digital simulations using morphologically accurate 3D head models based on real anthropometric data. These conditions represent an idealized scenario; therefore, the reported error values should be interpreted as a theoretical lower bound. In this study, practical aspects such as preparation time, ease of use, and cognitive load on technicians were not evaluated. In addition, factors like head movement, hair interference, and operator eye–hand coordination were not modeled, although they are expected to introduce further variability in real‐world applications. However, as these factors would likely affect all methods in a similar manner, they do not compromise the internal validity of the comparative analysis. The present study is therefore focused exclusively on the intrinsic geometric accuracy of each method.

BeamF3 is widely used due to its simplicity and applicability without imaging guidance. However, it has been shown to produce variable errors depending on cranial morphology (Fabregat‐Sanjuan et al. 2022; Mir‐Moghtadaei et al. 2015). Our findings confirm that BeamF3 yields a mean localization error exceeding 5.5 mm, with larger deviations observed in subjects whose cranial proportions deviate from population averages. These results underscore a key limitation of BeamF3; its fixed assumptions do not adequately accommodate anatomical variability. Consistent with this, recent MRI‐based modeling of BeamF3 targeting has demonstrated substantial interindividual variation in the cortical targets obtained from the same scalp‐based approach, highlighting the influence of individual anatomy on the resulting stimulation site (Rajasekharan et al. 2025).

The adjusted version of BeamF3 (Mir‐Moghtadaei et al. 2015) was designed to approximate an MRI‐derived DLPFC target rather than the F3 position defined by the International 10–20 system. Therefore, the distance measured in the present study should not be interpreted as an error in targeting its intended cortical target, but rather as the geometric distance between the Adjusted BeamF3 position and the standard F3 coordinate. The observed mean distance of over 8 mm indicates that the Adjusted BeamF3 position differs substantially from the standard F3 coordinate, while the relatively high standard deviation (1.7–1.8 mm) highlights considerable interindividual variability in this distance across subjects. Since the intended DLPFC target is based on MRI‐derived anatomy, its targeting accuracy cannot be assessed in the present study without participant‐specific neuroimaging data.

The SGP method, which utilizes proportional relationships between cranial landmarks (Jiang et al. 2022), demonstrated substantially improved accuracy and lower variability compared to both BeamF3 methods. Our results show that the SGP method achieved a mean localization error of approximately 2.5 mm, with a tighter error distribution. These findings align with those reported in the original SGP study, which cited an average technician accuracy of 4.1 ± 2.51 mm across scalp locations. In contrast, our analysis—free from operator error—reveals the theoretical minimum error of 2.39 mm for SGP.

The NeurallyF3 and EPlacementF3, yielded the highest localization accuracy, with mean errors below 0.3 mm. NeurallyF3 follows the same clinical methodology as BeamF3 but refines the *X* and *Y* proportions based on regression modelling (based on cranial landmarks), resulting in significantly improved accuracy and precision. EPlacementF3 further enhances accuracy by introducing a novel geometric framework and regression‐based prediction, using three cranial dimensions. While both methods achieved sub‐millimetric errors, EPlacementF3 outperformed NeurallyF3 in terms of mean, minimum, and maximum errors.

A major strength of NeurallyF3 and EPlacementF3 is their incorporation of multiple independent anatomical variables—HC, Ns–In, and Tr–Tr distances—into their prediction models. This multi‐variable approach enables better individualized localization, resulting in both higher accuracy and lower variability across diverse head shapes. These results demonstrate that these two methods outperform all others evaluated, not only in terms of mean localization error, but also in the consistency of performance across subjects. Notably, multiple linear regression shows that a large HC combined with a small Ns‐In, increases both values of %Ns to F3 and %Cz to C3’, while Tr‐Tr length has only a minor effect. In contrast, a simple linear regression of HC alone suggests the opposite trend, highlighting the importance of considering all relevant variables simultaneously, as analyzing HC in isolation could lead to misleading conclusions (Supplementary Information S1).

A key distinction between these two methods lies in their scope of applicability. NeurallyF3 is specifically designed for localizing the F3 position, as it follows the clinical procedure from the BeamF3 method (Pàmies‐Vilà et al. 2026), which targets this single point. In contrast, the underlying approach of EPlacementF3 can be generalized to other positions within the 10–20 and 10–10 systems. By applying the same methodology described in this study it is possible to extend EPlacementF3 to systematically estimate the location of all standard electrode positions. This scalability makes EPlacement a strong candidate for integration into device‐based solutions, offering the potential to automate and streamline coil placement in clinical and research settings.

Recent work in rTMS has increasingly emphasized the transition from standardized targeting approaches toward individualized neuromodulation. Jiang et al. (2025) highlighted the limitations of conventional non‐neuronavigated approaches, including the 5‐cm rule and BeamF3, particularly regarding their ability to account for interindividual anatomical variability. The authors emphasize the growing importance of individualized target localization to improve the anatomical precision and consistency of rTMS targeting. Recent studies (Valter et al. 2026a) show that standard templates like MNI152 do not accurately reflect correctly the average morphology of human brain, due to population‐specific biases, highlighting that individualized approaches may provide more accurate alternatives. Similarly, recent work (Riis et al. 2025) has shown that MRI‐free neuronavigation approaches using subject‐specific anatomical facial landmarks can approximate individual MRI‐based methods. Extending this line of evidence, a recent study demonstrates that deep brain targets can be localized using only scalp‐based measurements, without reliance on MRI or neuronavigation (Valter et al. 2026b). Further supporting this approach, another recent study shows that scalp‐based measurements derived from the standard 10–20 system are sufficient to infer the location of deep brain structures with sub‐4 mm accuracy, validating the 10–20 system as an effective bridge between external cranial anatomy and internal brain targets (Nafziger et al. 2025). Together, these findings reinforce the practical value of individualized, anatomy‐based methods as accurate, low‐cost alternatives to more complex and expensive methods such as MRI and optical neuronavigation methods.

Despite these promising results, real‐world use of any scalp‐based targeting method is subject to additional sources of error, including measurement inaccuracies, operator variability, and patient movement. Therefore, while the reported errors reflect optimal performance under ideal conditions, clinical validation is essential to confirm the practical utility and generalizability of these methods. Future studies should aim to assess performance across diverse populations, different age groups, and clinical settings.

A clear limitation of this study is that its analysis of skull–cortex relationships are based exclusively on adults, making the findings unsuitable for application to newborns and young children. In early development, the brain's anterior flexure, immature cortical folding, and rapidly changing skull morphology led to far poorer correspondence between external cranial landmarks and underlying cortical regions than in adults (Fu and Richards 2021; Tsuzuki et al. 2017; Zhang et al. 2021).

An additional conceptual limitation should be acknowledged. In this study, the International 10–20 F3 position was treated as the geometric reference (“true F3”). However, it is well established that the 10–20 F3 coordinate does not necessarily correspond to the exact anatomical location of the left DLPFC target to stimulate. Therefore, improving geometric accuracy relative to the 10–20 system does not automatically guarantee improved neuroanatomical targeting of the DLPFC. Nevertheless, the primary aim of this work was to reduce morphology‐related error in reproducing predefined scalp targets. Importantly, the proposed regression‐based framework—particularly the EPlacementF3 approach—is not inherently restricted to F3. The same methodology can be applied to any clinically defined scalp coordinate once a consensus anatomical target is established. At present, one of the challenges in NIBS is precisely the absence of a universally agreed and externally measurable definition of the optimal DLPFC target. In this context, improving the reproducibility and anatomical consistency of any selected scalp reference point represents a necessary step toward more standardized targeting.

## 5 Conclusions

This study highlights the limitations of traditional heuristic methods—BeamF3 and Adjusted BeamF3—for localizing the F3 position. BeamF3 method yielded mean errors of 5.59 ± 1.23 mm (range: 3.33–8.16 mm) in females and 6.09 ± 1.51 mm (range: 3.53–9.43 mm) in males. The adjusted version, intended to better align with the DLPFC, performed worse, with mean errors of 8.12 ± 1.96 mm for females and 8.96 ± 2.16 mm for males. These findings indicate that commonly used heuristic methods do not achieve precise localization of the true F3 point in anatomically diverse individuals. The SGP method demonstrated better accuracy, with a consistent mean error of 2.39 mm for both males and females and notably reduced variability. These findings demonstrate that neither heuristic method used until now achieved accurate localization of the true F3 point in any of the analyzed head models.

Alternatively, the NeurallyF3 achieves mean errors of just 0.19 ± 0.11 mm (range: 0.01–0.59 mm) in females and 0.23 ± 0.14 mm (range: 0.03–0.64 mm) in males in the validation dataset. Even more accurate, the EPlacementF3 method exhibited a small positioning error of 0.16 ± 0.12 mm (range: 0.01–0.59 mm) in females and 0.18 ± 0.11 mm (range: 0.03–0.47 mm) in males. Applying multiple linear regression model and considering the variability of cranial morphology, the new methods provide a highly accurate solution for determining the F3 position, with errors consistently below 1 mm.

These results represent a significant advance in the localization of F3 and potentially other 10–20 positions, addressing the anatomical variability that compromises current heuristic approaches. By offering a precise, scalable, and non‐invasive alternative to MRI‐guided targeting, the EPlacementF3 method, in particular, opens new possibilities for accurate and accessible stimulation site localization in clinical and research settings—especially in rTMS and other NIBS applications.

Future work should focus on clinical translation, integrating these models into easy‐to‐use tools or devices, and validating their performance across broader populations under realistic operational conditions.

## Author Contributions

**Rosa Pàmies‑Vilà**: conceptualization, methodology, investigation, writing – original draft. **Laia Mallol‑Bordas**: investigation, data analysis, writing – original draft. **Vicenç Pascual‑Rubio**: methodology, validation, writing – review and editing. **Albert Fabregat‑Sanjuan**: conceptualization, supervision, methodology, writing – review and editing.

## Funding

This research was funded by the 'la Caixa' Foundation (contract LCF/TR/CC22/52500009), by Instituto de Salud Carlos III (ISCIII) (Grant DTS24/00069) and co‐funded by the European Union, and from Agència de Gestió d'Ajuts Universitaris i de Recerca (AGAUR) Innovadors project (2025 INNOV 00056).

## Ethics Statement

This study did not involve human participants or animals. Analyses were conducted using computational simulations based on anatomically realistic head models derived from anthropometric datasets.

## Consent

The authors have nothing to report.

## Conflicts of Interest

The authors A. Fabregat‐Sanjuan and V. Pascual Rubio are inventors of the patented technology described in European Patent EP4337085B1, which relates to methods and systems for cranial localization and neurophysiological applications. The authors affirm that this intellectual property has not influenced the design, analysis, interpretation, or reporting of the research presented in this manuscript. All results and conclusions are reported objectively and transparently. Any potential financial benefits arising from the commercialization or licensing of the patent are managed according to the policies of the authors’ affiliated institutions. The remaining authors declare no conflicts of interest.

## Supporting information




**Supplementary Material**: brb371805‐sup‐0001‐SuppMat.docx

## Data Availability

The data that support the findings of this study are available from the corresponding author (a.fabregat@urv.cat) but restrictions apply to the availability of these data, which were used under research license from HumanShape project (University of Michigan) for the current study, and so are not publicly available. Data are, however, available from the authors upon reasonable request and with permission from HumanShape project (University of Michigan).
