# Supplementary material for: Reducing Cranial Morphology Error in F3 Scalp Localization: Comparison of Heuristic and Regression‐Based Methods
Source: Brain Behav. 2026 Oct 8;16(10):e71805. doi: 10.1002/brb3.71805 (PMC13647494; doi:10.1002/brb3.71805)
Supplement: Supplementary file 1 [file BRB3-16-e71805-s001.docx]

**SUPPLEMENTARY MATERIAL** from the publication “*Reducing cranial morphology error in F3 scalp localization: comparison of heuristic and regression-based methods”*

1. ***Supplementary analysis of multiple and simple linear regression model***

To complement the results shown in the main text, Supplementary Figures 11–14 display the multiple linear regression together with the corresponding simple linear regressions for each variable. These additional plots facilitate a more detailed evaluation of the individual effects of the predictors and help to clarify their relative influence on the values %Ns to F3 and %Cz to C3’.

In particular, the simple regression of HC shows an opposite trend compared to its effect in the multiple linear regression, whereas Ns-In and Tr-Tr exhibit consistent trends but with smaller slopes, indicating a weaker influence when considered individually. This highlights that the combined effect of a large HC together with a small Ns-In leads to an increase in the values of %Ns to F3 and %Cz to C3’, while HC alone is associated with decreases in both percentage values as HC increases.

*Figure 11 Effect of HC, Ns-In, and Tr-Tr on %Ns to F3: comparison between multiple linear regression (green) and simple linear regressions (blue). All regressions are centered at the mean value of each predictor. The Y-axis is scaled from −2σ to +2σ around the mean percentage. – FEMALE model*

*Figure 12 Effect of HC, Ns-In, and Tr-Tr on %Cz to C3’: comparison between multiple linear regression (green) and simple linear regressions (blue). All regressions are centered at the mean value of each predictor. The Y-axis is scaled from −2σ to +2σ around the mean percentage. – FEMALE model*

*Figure 13 Effect of HC, Ns-In, and Tr-Tr on %Ns to F3: comparison between multiple linear regression (green) and simple linear regressions (blue). All regressions are centered at the mean value of each predictor. The Y-axis is scaled from −2σ to +2σ around the mean percentage. – MALE model*

*Figure 14 Effect of HC, Ns-In, and Tr-Tr on %Cz to C3’: comparison between multiple linear regression (green) and simple linear regressions (blue). All regressions are centered at the mean value of each predictor. The Y-axis is scaled from −2σ to +2σ around the mean percentage. – MALE model*

2. ***Steps and workflow with FreeCAD***

**Localization of the F3 Point According to the International 10–20 System**

To determine the location of the F3 position according to the International 10–20 system, the following procedure was performed:

1. A straight line was drawn between the Nasion (Ns) and Inion (In) along the sagittal plane of the head.
2. The Cz position was defined as the midpoint of the Ns–In distance.
3. Eleven reference points were identified along the Ns–In axis (Figure 15), dividing the total Nasion–Inion distance into 10% increments. Among these points (yellow in Figure 15), Fpz, Fz, and Oz correspond to the 10%, 30%, and 90% positions along the axis, respectively.

*Figure 15 Nasion (Ns) - Inion (In) along the sagittal plane with 10% increments (yellow points).*

4. A plane was generated between the left and right Tragus points, passing through Cz. Its intersection with the head surface defined the Tragus–Tragus line.
5. A second plane was constructed through Fpz and Oz (Figure 16), together with the point at which the Fpz–Oz axis intersects the Tragus–Tragus line, referred to as the HP point. This plane was used to define the cranial perimeter.
6. The cranial perimeter was obtained by calculating the intersection between this plane and the head mesh.

*Figure 16 Tragus–Tragus line and cranial perimeter plane.*

7. The positions of F7 and F8 were then determined along the cranial perimeter at 85% and 15%, respectively, of the total perimeter distance measured from Fpz.
8. Finally, a plane passing through F7, F8, and Fz was created. The F3 position was subsequently defined as the midpoint between F7 and Fz (Figure 17).

*Figure 17 Line passing through F7, F8, and Fz. The F3 position was subsequently defined as the midpoint between F7 and Fz.*

**Localization of the Beam-F3 Point**

For the determination of the Beam-F3 position, steps 1–6 of the International 10–20 procedure were followed to obtain the Tragus–Tragus, Nasion–Inion, and cranial perimeter measurements.

These measurements were then used as input parameters for the Beam algorithm, which was implemented to calculate the two distances required to determine the Beam-F3 position, X and Y. For the Adjusted Beam-F3 position, an additional 0.35 mm was incorporated into the corresponding calculation.

The following procedure was subsequently applied:

1. A reference point, designated F3X, was positioned at a distance X measured clockwise from Fpz along the cranial perimeter (Figure 18a).
2. A plane passing through F3_X, Cz, and HP was then constructed. The intersection of this plane with the head mesh defined the trajectory along which the Beam-F3 position was located.
3. Finally, the Beam-F3 point was positioned at a distance Y from Cz along the trajectory defined in the previous step (Figure 18b).

*Figure 18 Beam-F3 position. Left (18a) shows a yellow point (F3_X) indicating distance X measured clockwise from Fpz along the cranial perimeter. Right (18b) shows Beam-F3 point positioned at a distance Y from Cz along the trajectory defined by Cz – F3_X line.*

**Description of the FreeCAD Python Workflow**

The lines used to determine the F3 position according to the EPlacementF3, NeurallyF3, and SGP methods are shown in previous figures. The Python implementation developed in FreeCAD is a common workflow for the EPlacementF3, NeurallyF3, and SGP methods as follows:

1. **Define anatomical reference points.**
   The required anatomical landmarks are identified on the three-dimensional head mesh.
2. **Construct reference planes.**
   Planes are defined using three reference points according to the geometric requirements of each F3 localization method.
3. **Calculate plane–mesh intersections**.
   The intersections between each reference plane and the head mesh generate curves corresponding to trajectories along the scalp surface.
4. **Generate continuous scalp trajectories.**
   When necessary, successive curve segments are joined to obtain a continuous wire between the required reference points.
5. **Discretize each trajectory.**
   Each wire is converted into a path array, providing a high-resolution representation of the scalp trajectory.
6. **Calculate distances and relative positions.**
   The path arrays are used to calculate accumulated distances between reference points and to express specific positions as percentages of the total trajectory length.
7. **Generate method-specific reference points.**
   When required, trajectories are divided into predefined intervals. For example, the Nasion–Inion trajectory is divided into 10% increments, whereas the Fz–F7 trajectory is represented by five reference points
8. **Determine the F3 position.**
   The coordinates and relative positions obtained from the previous steps are combined according to the geometric definition of each method to determine the final F3 location.

Although the geometric constructions differ between EPlacementF3, NeurallyF3, and SGP, all three methods are implemented using this common computational framework.
